# Supplementary material for: Widely Targeted Metabolic Profiling Reveals Differences in Polyphenolic Metabolites during Rosa xanthina f. spontanea Fruit Development and Ripening
Source: Metabolites. 2022 May 13;12(5):438. doi: 10.3390/metabo12050438 (PMC9147897; doi:10.3390/metabo12050438)
Supplement: Supplementary file 1 [file metabolites-12-00438-s001.zip › supplementary figure.pdf]

A

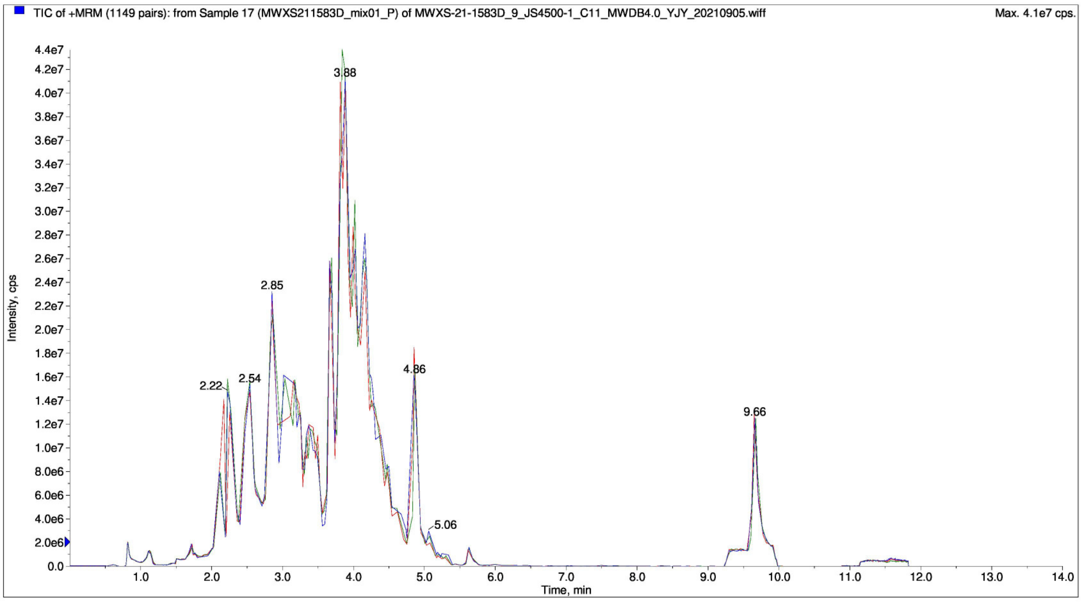

B

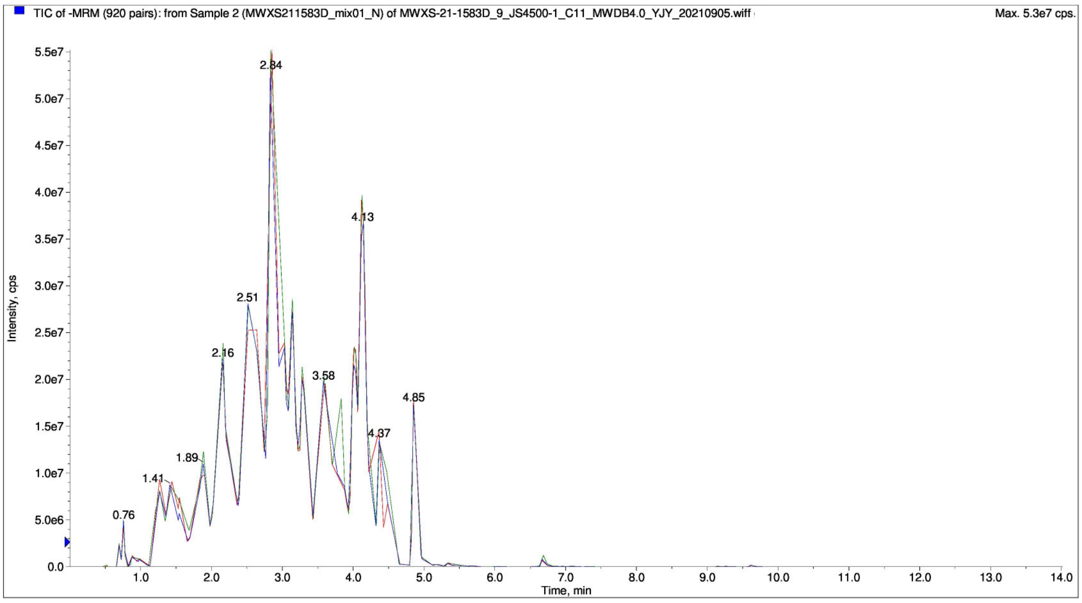

Figure S1 QC Sample Mass Spectrometry Detects TIC Overlapping Diagram

Note: Graph A is a positive ion mode, while graph B is a negative ion mode and. The abscissa is the retention time (RT) of metabolite detection, and the ordinate is the ion current intensity (CPS, count per second) of ion detection. The results show that the curves of total ion current detected by metabolites have high overlap, that is, the retention time and peak intensity are consistent, which indicates that the signal stability is good when the same sample is detected by mass spectrometry at different times.

A

B

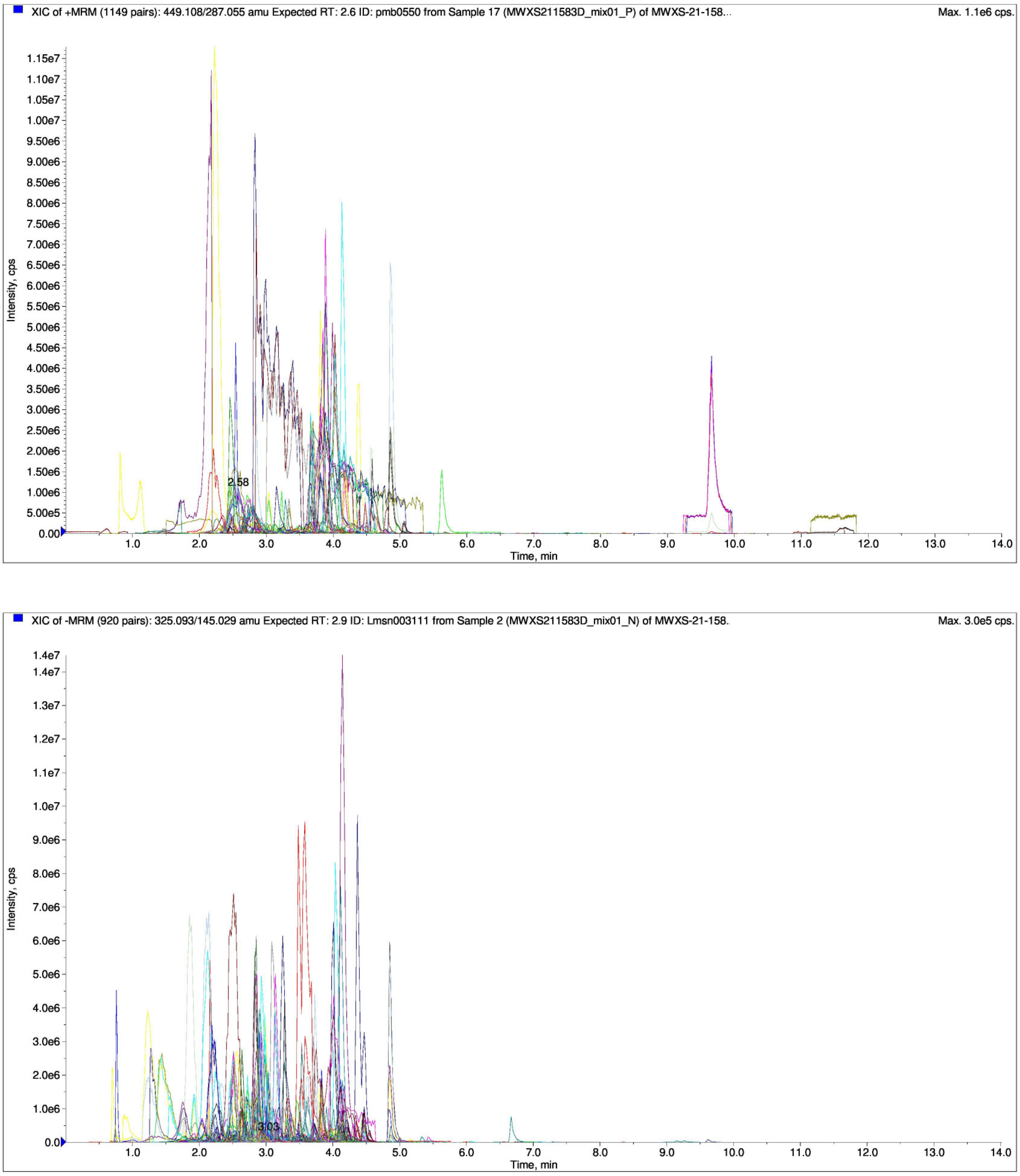

Figure S2 Multiple reaction monitoring (MRM) graph of the QC sample.

Note: Graph A is a positive ion mode, while graph B is a negative ion mode and each mass spectrum peak with different colors represents a metabolite detected. The abscissa is the retention time (RT) of metabolite detection, and the ordinate is the ion current intensity (CPS, count per second) of ion detection.
